# Transcriptional silencing of 35S driven-transgene is differentially determined depending on promoter methylation heterogeneity at specific cytosines in both plus- and minus-sense strands

**DOI:** 10.1186/s12870-019-1628-y

**Published:** 2019-01-14

**Authors:** Wataru Matsunaga, Hanako Shimura, Senri Shirakawa, Reika Isoda, Tsuyoshi Inukai, Takeshi Matsumura, Chikara Masuta

**Affiliations:** 1 Research Faculty of Agriculture, Hokkaido University, Kita 9 Nishi 9, Kita-ku, Sapporo, 060-8589 Japan; 2 Bioproduction Research Institute, National Institute of Advanced Industrial Science and Technology (AIST), 2-17-2-1 Tsukisamu-Higashi, Toyohira-ku, Sapporo, 062-8517 Japan

**Keywords:** CaMV 35S promoter, *Nicotiana benthamiana*, RNA-directed DNA methylation, VITGS

## Abstract

**Background:**

De novo DNA methylation triggered by short interfering RNAs is called RNA-directed DNA methylation (RdDM). Transcriptional gene silencing (TGS) through RdDM can be induced using a viral vector. We have previously induced RdDM on the 35S promoter in the green fluorescent protein (GFP)-expressing *Nicotiana benthamiana* line 16c using the cucumber mosaic virus vector. The GFP fluorescence phenotype segregated into two types, “red” and “orange” in the first self-fertilized (S_1_) progeny plants by the difference in degree of recovery from TGS on *GFP* expression. In the second self-fertilized generation (S_2_ plants), the phenotypes again segregated. Explaining what generates the red and orange types could answer a very important question in epigenetics: How is the robustness of TGS maintained after RdDM induction?

**Results:**

In bisulfite sequencing analyses, we found a significant difference in the overall promoter hypermethylation pattern between the red and orange types in S_1_ plants but little difference in S_2_ plants. Therefore, we assumed that methylation at some specific cytosine residues might be important in determining the two phenotypes. To find the factor that discriminates stable, robust TGS from the unstable TGS with incomplete inheritance, we analyzed the direct effect of methylated cytosine residues on TGS. Because it has not yet been demonstrated that DNA methylation at a few specific cytosine residues on known sequence elements can indeed determine TGS robustness, we newly developed a method by which we can directly evaluate the effect of specific methylation on promoter activity. In this assay, we found that the effects of the specific cytosine methylation on TGS differed between the plus- and minus-strands.

**Conclusions:**

We found two distinct phenotypes, the stable and unstable TGS in the progenies of virus-induced TGS plants. Our bisulfite sequencing analyses suggested that methylation at some specific cytosine residues in the 35S promoter played a role in determining whether stable or unstable TGSs are induced. Using the developed method, we inferred that DNA methylation heterogeneity in and between the plus- and minus-strands can differentially determine TGS.

**Electronic supplementary material:**

The online version of this article (10.1186/s12870-019-1628-y) contains supplementary material, which is available to authorized users.

## Background

DNA methylation is a highly conserved epigenetic hallmark in plant genomes that controls gene expression. Regulation of gene expression by transcriptional gene silencing (TGS) of transgenes and endogenous genes has been extensively studied [1–4]. In plants, DNA methylation occurs in three sequence contexts: CG, CHG and CHH (where H is A, C or T). The CG methylation is maintained by METHYLTRANSFERASE1 (MET1), whereas CHG and CHH methylation is maintained by plant-specific CHROMOMETHYLASE3 (CMT3) and CHROMOMETHYLASE 2 (CMT2), respectively [1–5]. In addition, the de novo DNA methyltrasferase, DOMAINS REARRANGED METHYLTRANSFERASE2 (DRM2) is involved in both maintenance and initiation of DNA methylation [6].

In plants, DRM2 is required for de novo DNA methylation, which is triggered by short-interfering RNAs (siRNAs) [6]. siRNAs guide DRM2 to the target sequences on the genomic DNA and directs de novo DNA methylation through RNA-directed DNA methylation (RdDM) [7–18], in which two plant-specific RNA polymerases, Pol IV and Pol V play essential roles. The 24-nucleotide (nt) siRNAs are generated and amplified by Pol IV, RNA-DEPENDENT RNA POLYMERASE 2 (RDR2) and DICER-LIKE 3 (DCL3). The 24-nt siRNAs are then loaded onto Argonaute (AGO) for the subsequent DRM2-mediated DNA methylation. AGO4 eventually interacts with the Pol V subunit, NUCLEAR RNA POLYMERASE E1 (NRPE1) and makes a complex with DRM2 to initiate DNA methylation. Recently, RdDM has been classified into two pathways, canonical and non-canonical. In the canonical pathway, a cascade of enzyme reactions (Pol IV-RDR2-DCL3) produces 24-nt siRNAs in concert, whereas several additional small RNAs seem to be involved in RdDM through the non-canonical pathway [19].

Assuming that RdDM is triggered by siRNAs from a promoter sequence, there are two options to induce specific DNA methylation artificially: transgene-induced or virus-induced. The first involves the creation of transgenic plants where double-stranded RNAs (dsRNAs) against the target promoter sequence are generated, for example, by an inverted repeat construct [20–22], while the second involves the virus-induced transcriptional gene silencing (VITGS), which is quite convenient because we do not have to produce transgenic plants [23]. However, there is a large difference between the two methods in that transgene-induced siRNAs are generated even in the progeny plants but virus-driven siRNAs are not detectably carried into the next generation without viral infection.

A substantial number of viruses has been developed for VITGS, e.g., apple latent spherical virus (ALSV) [24–26], tobacco rattle virus [27, 28], barley stripe mosaic virus [29] and potato virus X [30, 31]. We have previously developed the cucumber mosaic virus (CMV)-based vector, CMV-A1 for both PTGS and TGS [32–37]. Using CMV-A1, we could successfully induce VITGS against some endogenous genes as well as a transgene [33, 38]. When we inoculated the green fluorescent protein (GFP)-expressing transgenic *Nicotiana benthamiana* (16c) with the CMV-A1 constructs containing various sizes of the 35S promoter sequences, we found that the *GFP* expression levels were downregulated due to RdDM and that the sizes of the virus-integrated sequences were important for DNA methylation [39].

The CaMV 35S promoter is such a strong promoter that it has been widely used for gene expression in plants. Several sequence domains affecting promoter activity have already been identified. The activation sequence factors 1 and 2 (*as-1* and *as-2*), which are located in subdomains A1 and B1, respectively, have been found as a transcription factor-binding site [40–45]. Subdomains B2 and B4 have been also reported to be important for the promoter activity [46]. However, it has not yet been demonstrated that specific DNA methylation on those sequence elements can control the promoter activity.

In our observation of the VITGS against the 35S promoter in 16c plants, we noticed that the virus-free S_1_ progeny plants comprised two phenotypes when exposed to UV light: red coloration (RED) resulting from autofluorescence of chlorophylls due to loss of GFP fluorescence by TGS and orange coloration (ORN) from the combined chlorophyll autofluorescence and GFP fluorescence. Most of the S_2_ progeny plants from the S_1_ ORN plants were ORN plants, but occasionally RED plants appeared. Virus vector-derived siRNAs corresponding to the 35S promoter sequence would not be generated in the progeny plants because progenies are no longer infected by the recombinant virus. We raise the following questions regarding unknown mechanisms for maintenance and initiation of the DNA methylation: What discriminates the RED and ORN plants? How are stable and unstable TGS controlled? Our preliminary analyses of the overall DNA methylation status of the 35S promoter in the two phenotypes indicated that there was not much difference between the RED and ORN plants, suggesting that methylation at specific cytosine residues on the target sequence may be important for inheritance of TGS. To answer the above questions, we here analyzed the DNA methylation in our VITGS-induced 16c plants in detail.

## Results

### Differential induction of VITGS depending on the sizes of target sequences

**Fig. 1:**
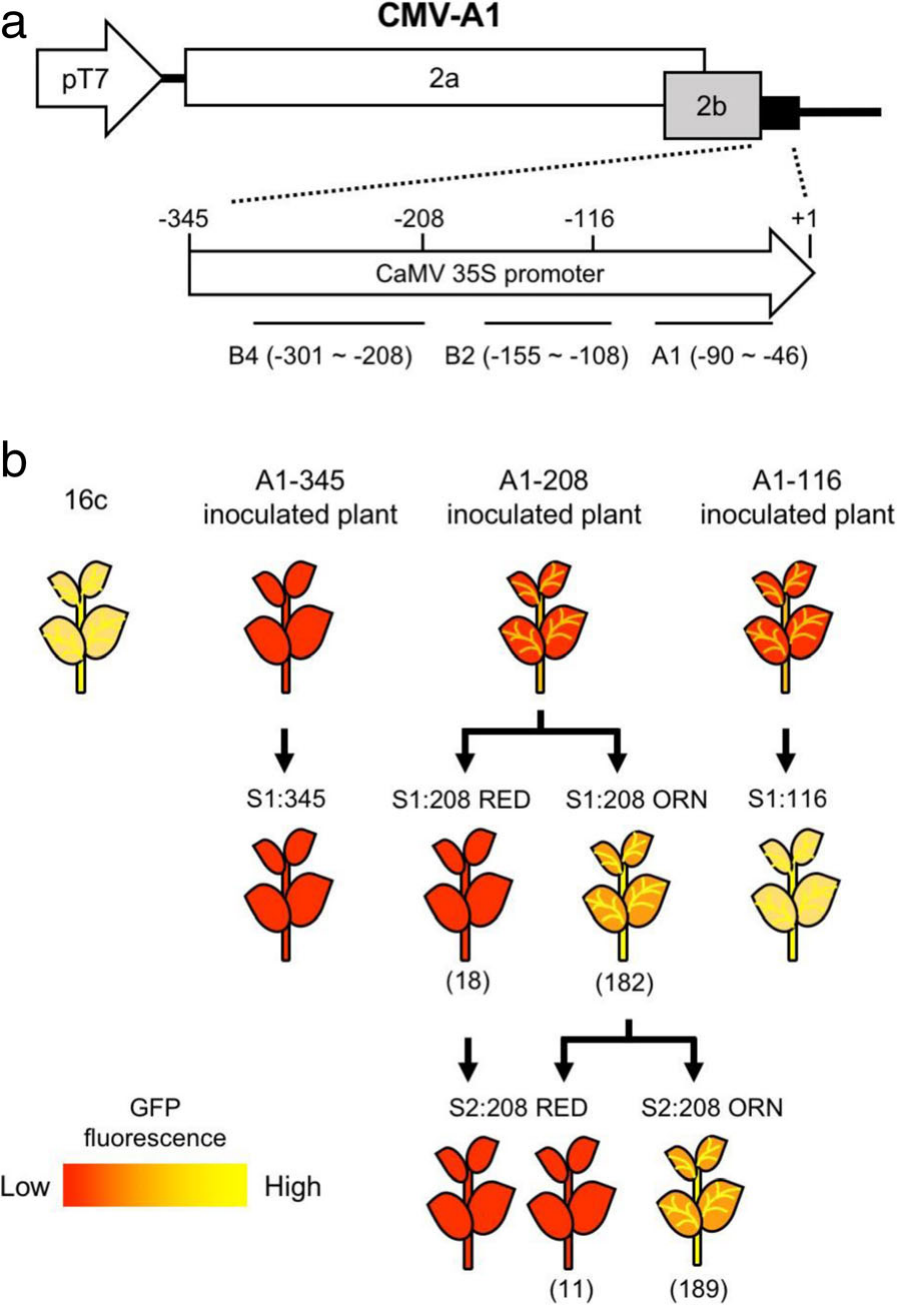
CMV vector constructs and illustrations of phenotypes of the virus-infected plants and its progenies. **a** Diagram of CMV-A1vector constructs. The recombinant CMV-A1 vectors contained the CaMV 35S promoter segments of three different sizes (positions relative to the transcription start site − 345 to + 1, − 208 to + 1 and − 116 to + 1). A1, B2 and B4 are sequence domains that affect promoter activity of the CaMV 35S sequences. **b** Schematic illustration of virus-infected plants and its progenies. *Nicotiana benthamiana*16c plants were inoculated with CMV-A1 vectors containing a CaMV 35S promoter fragment (A1–345, A1–208 or A1–116), and then first (S_1_) and second (S_2_) self-fertilized progenies were obtained. The phenotype segregation is indicated in parenthesis. The red color represents appearance of chlorophyll autofluorescence as a consequence of the loss of GFP fluorescence by TGS, while the yellow color represents combined expression of chlorophyll autofluorescence and GFP fluorescence

**Fig. 2:**
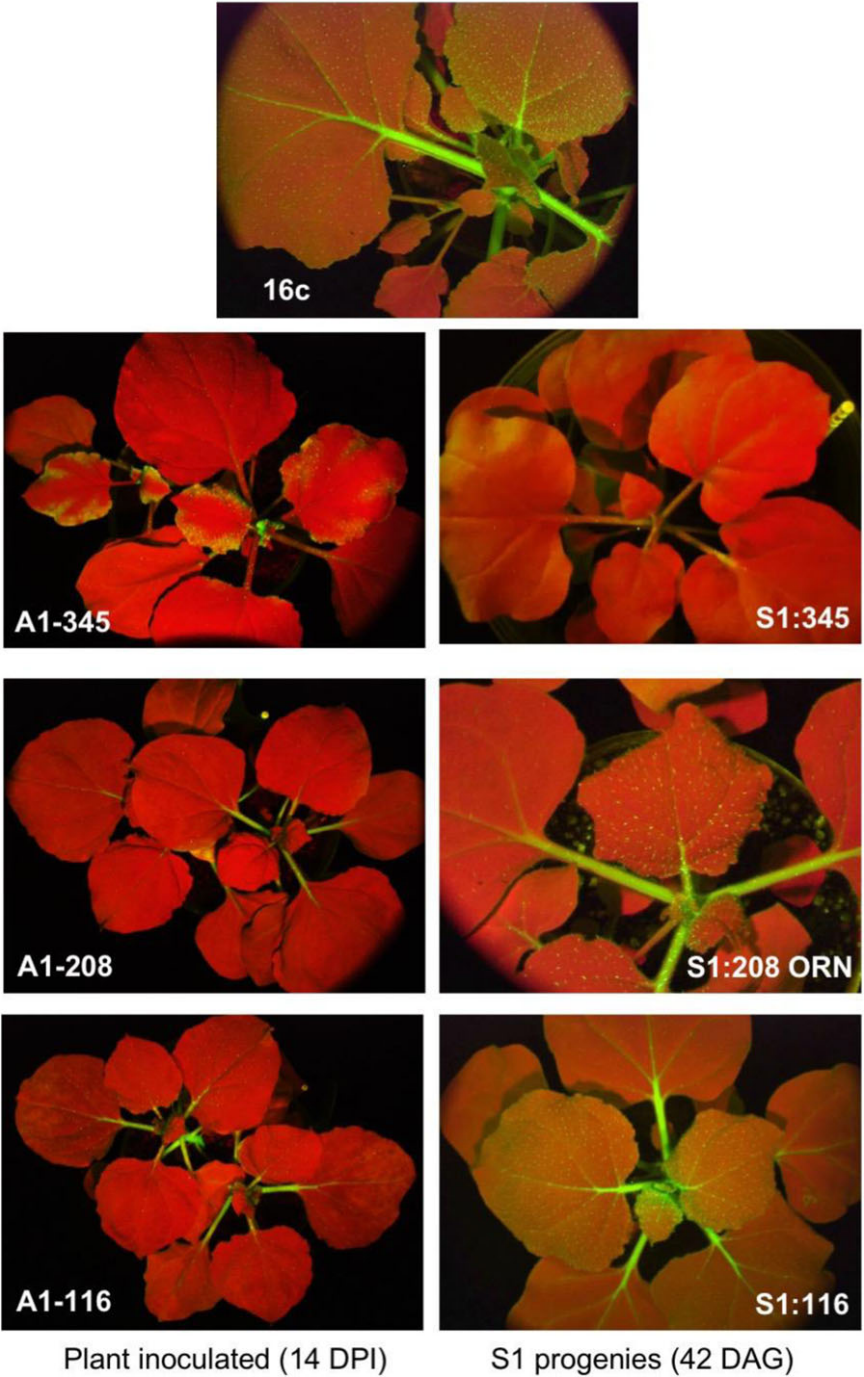
GFP fluorescence of the plants inoculated with the CMV-A1 constructs and their S_1_ progenies. The GFP-expressing 16c plants were inoculated with the CMV-A1 vectors containing a CaMV 35S promoter fragment (A1–345, A1–208 or A1–116). Photographs were taken at 14 days post inoculation (DPI) for the inoculated plants and 48 days after germination (DAG) for the S_1_ plants

We previously induced TGS of the 35S promoter in GFP-expressing *N. benthamiana* plants (16c) by the CMV-A1 vector (Fig. 1a). In this study, we further characterized this VITGS from a mechanistic viewpoint. Depending on the sizes of the 35S promoter sequences integrated into the viral vector, different degrees of TGS induction were found. When the 345-nt fragment corresponding to almost the entire 35S promoter was cloned, the virus-infected plants developed efficient VITGS, which was well maintained in the first self-fertilized (S_1_) progeny plants (S1:345) that fluoresced red under UV light (Figs. 1b and 2). The red-color must be due to chlorophyll autofluorescence because GFP expression was greatly reduced. On the other hand, the 116-nt fragment could induce TGS in the inoculated plants, in which weak GFP fluorescence still remained on the leaf veins, but failed to maintain the TGS in the S_1_ progeny plants (S1:116); GFP fluorescence in S1:116 plant was recovered to the level in 16c plant, yielding a yellow phenotype in leaves (Figs. 1b and 2). For the 208-nt fragment, we observed VITGS induction in the A1–208-inoculated plants, but the plants showed somewhat GFP fluorescence in the petiole of leaves. The S_1_ progeny plants from A1–208-inoculated plants segregated into two phenotypes: red type, which lost GFP fluorescence (designated S1:208 RED) and orange type, which was intermediate between S1:345 and 16c (designated S1:208 ORN) (Figs. 1b and 2). ORN plants seem to retain a certain level of GFP fluorescence, which masks the red autofluorescence. None of the S_1_ progeny plants from the A1–208-inoculated plants had fully recovered GFP fluorescence.

**Fig. 3:**
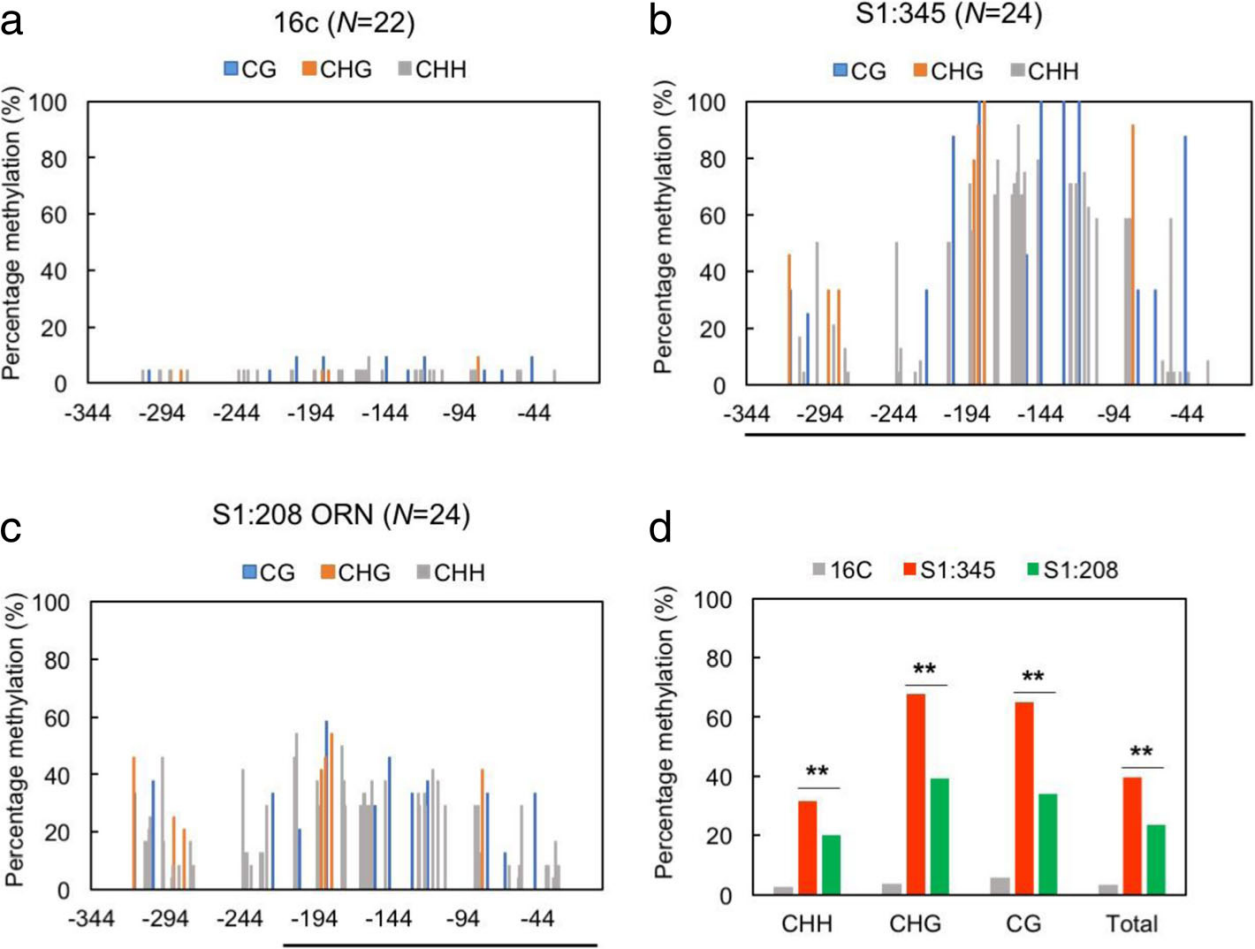
Cytosine methylation frequency in the 35S promoter of the S_1_ progenies. **a-c** Methylation frequency of plus-strand of the CaMV 35S promoter in S1:345, S1:208 ORN and the control 16c plants. *N* is the number of the clones used for the bisulfite sequencing. The *x*-axis is the position relative to the nucleotide distance from the transcription start sites. Bars under the *x*-axis in **b** and **c** are the nucleotide position of the CaMV 35S promoter sequence integrated in the CMV-A1 vectors. **d** Summary of the results (**a** to **c**) to show differences in CHH, CHG, CG and total methylation. The asterisks indicate a statistical significance in methylation frequencies between S1:345 and S1:208 ORN by two-tailed Fisher’s exact test (** *P* < 0.01)

Because VITGS is essentially operated through RdDM, we then investigated the methylation status on the 35S promoter in S1:345, S1:208 ORN and 16c plants. Our bisulfite sequencing analyses revealed that the methylation level of the entire 35S promoter was significantly higher in S1:345 than in S1:208 ORN, and that the 35S promoter in 16c had little methylation (Fig. 3).

### Analysis of methylation of 35S promoter in second-generation self-fertilized progenies from VITGS plants

**Fig. 4:**
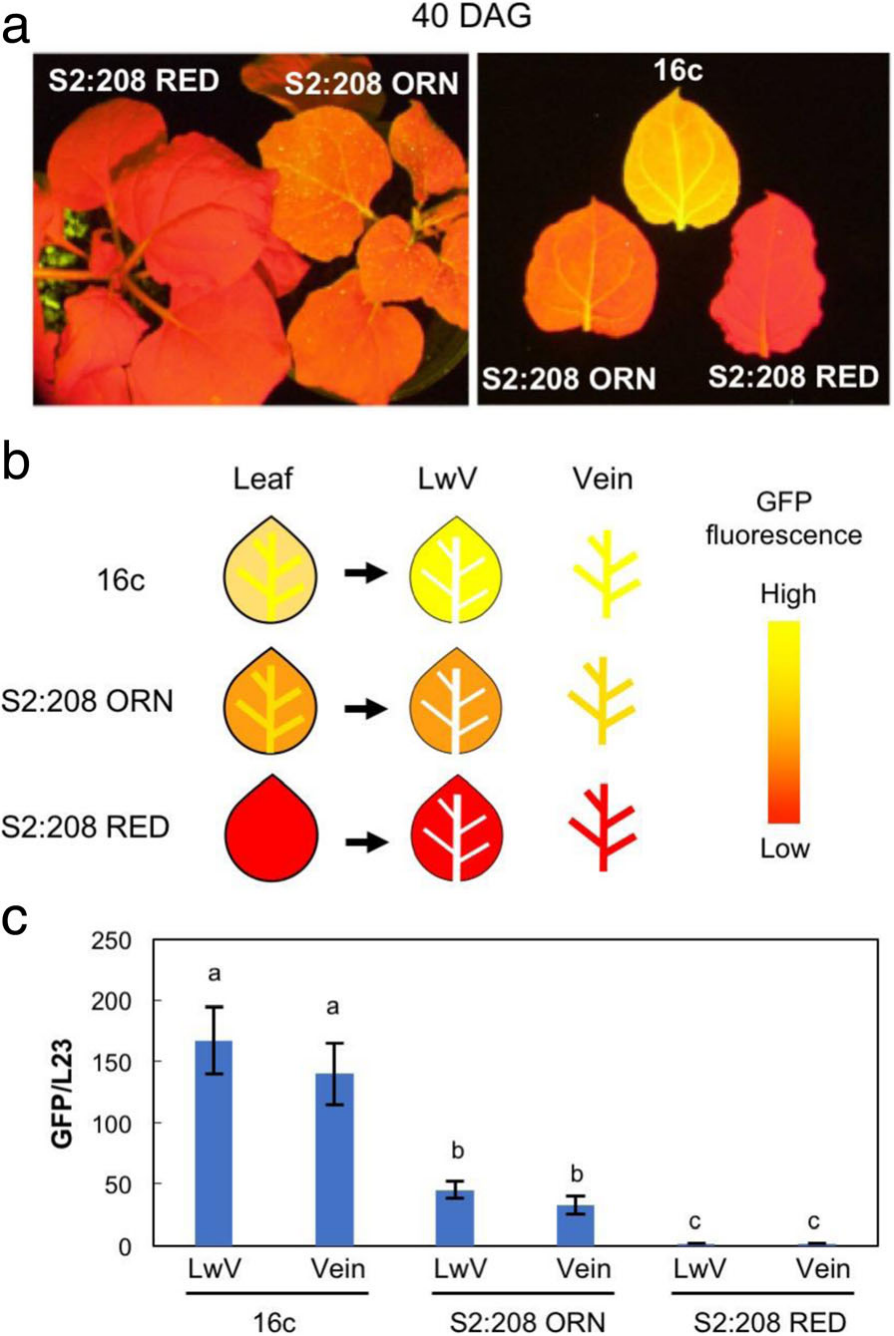
Segregated GFP fluorescence pattern in the S2:208 plants. **a** GFP fluorescence of the S2:208 RED and S2:208 ORN. Photographs were taken at 40 DAG. Red-colored fluorescence by chlorophyll autofluorescence was observed in S2:208 RED, while S2:208 ORN showed orange-colored fluorescence that were intermediate phenotype between 16c and S2:208 RED. **b** Schematic illustration of preparation of the leaf samples; leaf veins tissues (Vein) were separated from the other parts of leaf blade (leaf without veins, LwV) and used subsequent experiments. **c** Expression levels of the *GFP* gene in Vein and LwV tissues from S2:208 RED and S2:208 ORN. Values represent means of three biological replicates. Error bars represent standard deviations. Data were analyzed using the Tukey-Kramer test. Different lowercase letters on error bars indicate a significant difference at the 5% level between the means

When we produced S_2_ progeny plants by a second self-fertilization of the S1:208 ORN plants, the GFP fluorescence phenotype again segregated into RED and ORN plants: S2:208 RED and S2:208 ORN (Figs. 1b and 4a). When the S1:208 RED plant was self-fertilized, we observed only RED phenotype plants in the S_2_ progenies. These results raised questions: Does the stable, inherited TGS depend on the methylation level? What is the threshold methylation level for the stable TGS? However, because it is often the case that a few methylated cytosine residues can inhibit promoter activity, the stable TGS may not necessarily be explained only by a difference in hypermethylation. To find further clues, we scrutinized the intermediate TGS in S2:208 progenies.

Among S_2_ progenies, we found that the leaf veins (Vein) fluoresced brighter yellow than the other parts (leaf without vein, LwV) (Fig. 4a and b). We separated Vein and LwV and compared the *GFP* expression levels in those tissues by qRT-PCR (Fig. 4c). As we expected from the GFP fluorescence intensity, *GFP* was expressed in S2:208 ORN at a level of about 1/3 of that in 16c, while it was barely detected in S2:208 RED. On the other hand, unlike our expectation, the *GFP* level differed little between Vein and LwV in the samples.

**Fig. 5:**
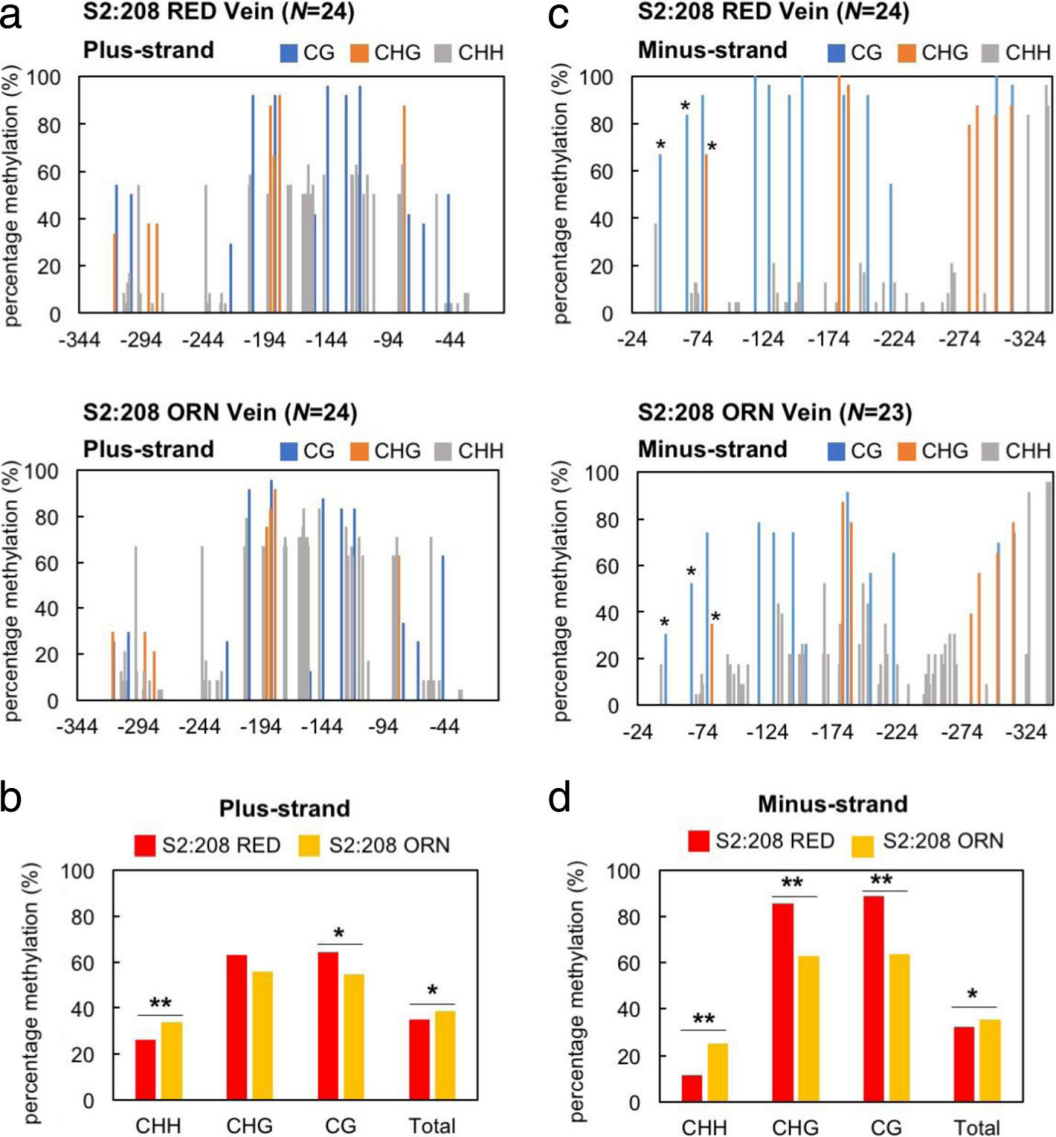
Cytosine methylation frequency in the 35S promoter in the Vein tissues of S2:208 RED and S2:208 ORN. **a** Comparison of methylation status in the plus-sense of 35S promoter between S2:208 RED and S2:208 ORN. **b** Summary of the results of **a** to show differences in CHH, CHG, CG and total methylation. **c** Comparison of the methylation status in the minus-sense sequence of the 35S promoter between S2:208 RED and ORN. Asterisks indicate cytosine residues that are significantly different in methylation frequency between RED and ORN as explained in Fig. 6b. **d** Summary of the results of **c** to show differences in CHH, CHG, CG and total methylation. *N* is the number of the clones used for the bisulfite sequencing. The *x*-axis shows the position relative to the transcription start site (+ 1). The asterisks in (**b**) and (**d**) indicate a statistical significance in methylation frequencies by two-tailed Fisher’s exact test (* *P* < 0.05, ** P < 0.01)

**Fig. 6:**
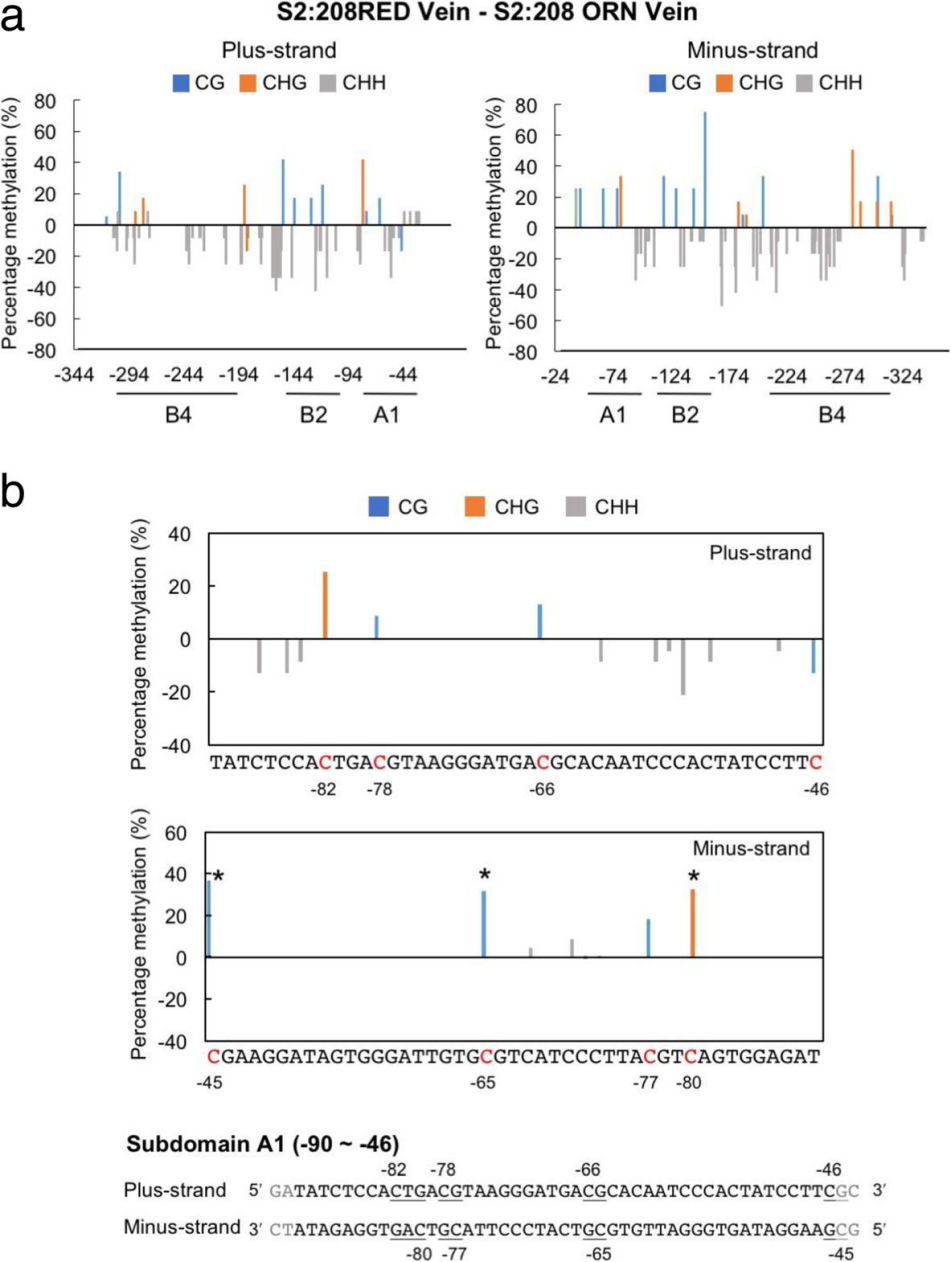
Difference in specific cytosine methylation between S2:208 RED and S2:208 ORN in the Vein tissues. **a** Difference in methylation frequencies in the overall plus- and minus-strand of the 35S promoter in S2:208 RED and S2:208 ORN. To make it easier to find any differences in methylation frequencies in each strand, values of S2:208 RED Vein (Fig. 5a and c, upper graphs) were subtracted from those of S2:208 ORN Vein (Fig. 5a and c, lower graphs). **b** Close-up of the subdomain A1 in **a**. The *x*-axis is the nucleotide sequence of the subdomain A1. Nucleotide sequences of the plus- and minus-strand of the subdomain A1 are indicated below the graph. Methylated CG and CHG sites are underlined. The asterisks in (**b**) indicate a statistical significance between S2:208 RED and S2:208 ORN by two-tailed Fisher’s exact test (* *P* < 0.05)

We next used bisulfite sequencing analyses to search for differences in the methylation pattern on the 35S promoter between S2:208 RED and ORN plants. As shown in Additional file 1: Figure S1, there were few methylations in the 35S promoter in 16c plants, and no large difference was observed in methylation status between Vein and LwV tissues. The overall DNA methylations on the plus-sense of the 35S promoter in S2:208 RED and S2:208 ORN were shown in Fig. 5 for Vein, and in Additional file 2: Figure S2 for LwV tissues. The total methylation in Vein tissues was higher in S2:208 ORN (Fig. 5b), although total methylation in LwV tissues was higher in S2:208 RED (Additional file 2: Figure S2b). On the other hand, we noticed that asymmetric methylation (CHH methylation) was significantly lower, but symmetric CG methylation was higher in the Vein of S2:208 RED than S2:208 ORN (Fig. 5b). Because asymmetric methylation on one strand does not occur on its complementary strand at the corresponding positions and is not inherited by progeny, we further analyzed the methylation status on the complementary, minus-strand. DNA methylations on the minus-strand of the 35S promoter were shown in Fig. 5c for Vein and in Additional file 2: Figure S2c for LwV. When the methylation status of minus-strand was statistically analyzed, symmetric (CG and CHG) methylations in S2:208 RED were significantly higher than S2:208 ORN in both Vein and LwV tissues (Fig. 5d and Additional file 2: Figure S2d). In addition, the overall CHH methylation was greatly reduced in S2:208 RED compared to S2:208 ORN. It is thus likely that symmetric methylation at specific sites and a decrease in CHH methylation may be correlated with the stable, inherited TGS in the VITGS progeny plants. To make it easier to find any differences in methylation frequency between RED and ORN, we subtracted the values in S2:208 ORN from those in S2:208 RED (Fig. 6 and Additional file 3: Figure S3). In the subdomain A1, the methylation levels of the cytosine residues involved in symmetric methylation (cytosines at − 45, − 65, − 77 and − 80) were significantly higher in S2:208 RED than in S2:208 ORN (Fig. 6b and Additional file 3: Figure S3b). Symmetric methylation in plus-sense tended to be higher at the specific cytosine (position − 82) in S2:208 RED than S2:208 ORN, although statistically not supported. These results suggest that methylation in the minus-strand would be more important than that in the plus-strand for stable TGS.

In parallel with the VITGS experiments, we were creating transgenic *Arabidopsis* plants to screen for plants that showed TGS of the *GFP* gene under the 35S promoter; we obtained a transgenic line that contained a direct repeat sequence of the 35S promoter fused to the *GFP* gene. In the progeny generations (T_1_ to T_5_) of the direct repeat line, we noted two types of GFP fluorescence: one showing GFP fluorescence at an early stage, then developed post-transcriptional gene silencing (PTGS) for *GFP* expression and eventually induced TGS at a late stage (PTGS-TGS plants); the other already had perfect TGS in small seedlings (TGS plants), and the TGS was stably inherited by the next generation. From the results of bisulfite sequencing to analyze the methylation status of the 35S promoter sequences in those plants, we found that CG or CHG methylations at positions − 46, − 66, − 78 and − 82 in the subdomain A1 were higher in the TGS plants than in the PTGS-TGS plants (Additional file 4: Figure S4). Furthermore, the overall CHH methylation was greatly reduced in the TGS plants compared to the PTGS-TGS plants. These observations therefore agree well with the results of VITGS.

### CG methylation at a few cytosine residues in the minus-strand of the 105-bp sequence containing the subdomain A1 caused significant reduction in promoter activity

**Fig. 7:**
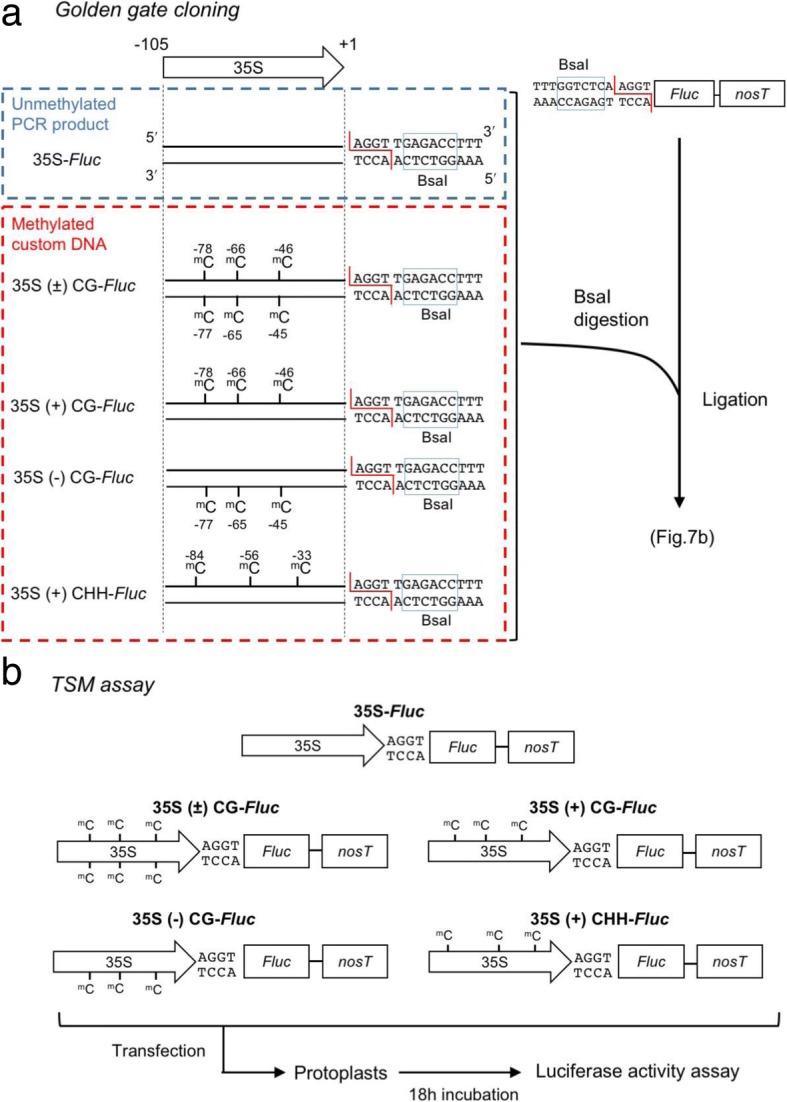
Strategy to assess the direct effect of strand-specific cytosine methylation on promoter activity. **a** Golden gate cloning to construct the firefly luciferase (*Fluc*) gene fused with a methylated 35S promoter sequence. Custom-synthesized DNAs containing three methylated cytosine residues (positions − 78, − 66 and − 46) at CG context and three methylated cytosine residues (positions − 84, − 56 and − 33) at CHH context were annealed, digested with BsaI and ligated to the BsaI-digested *Fluc* gene. **b** Schematic flow of transient expression assay in protoplasts. In vitro ligation products were directly transfected to *N. benthamiana* protoplasts, and then luciferase activities in 18 h-incubated protoplasts were measured by fluorometer

**Fig. 8:**
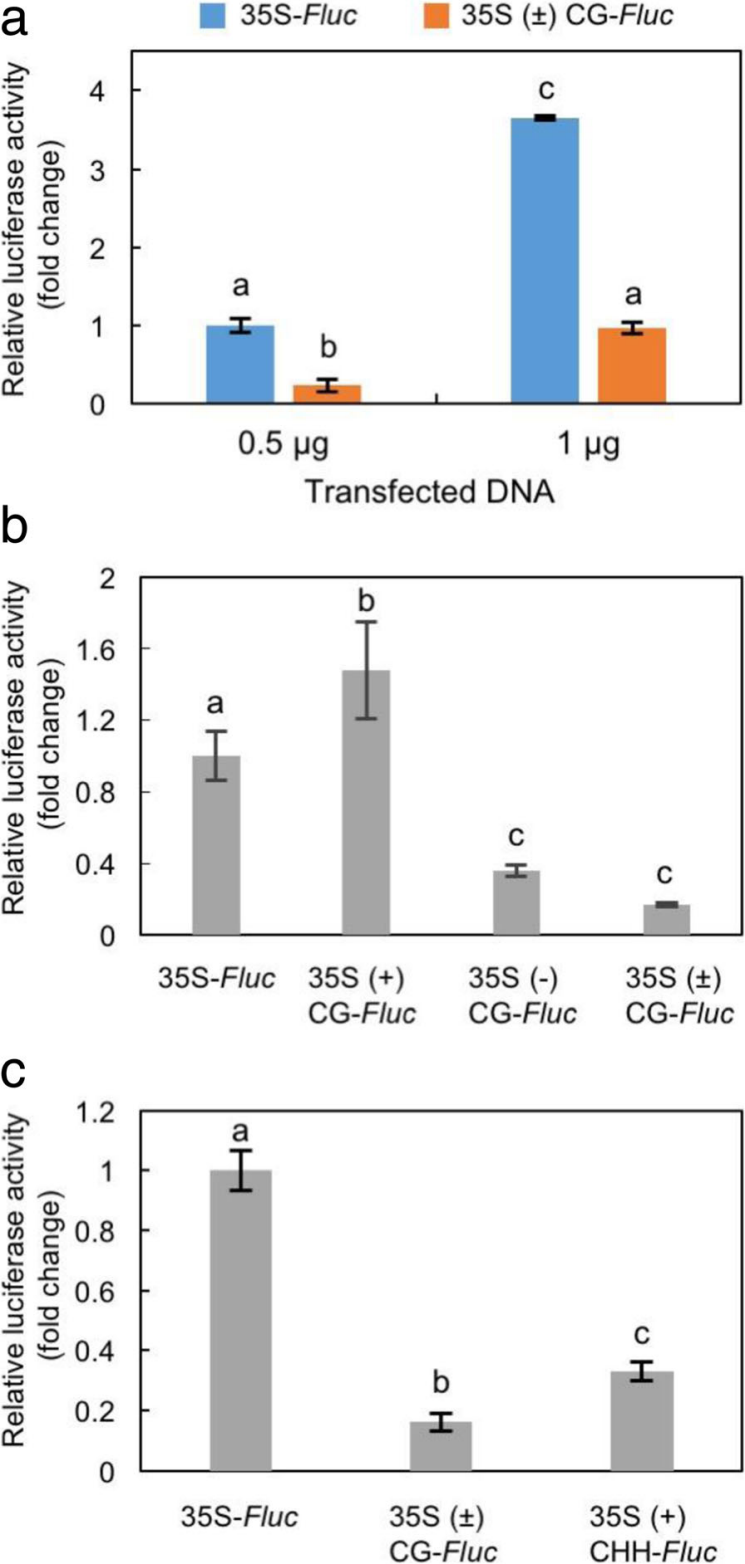
Effect of specific cytosine methylation in the 35S promoter on promoter activity. **a** Decrease of promoter activity of the 35S sequence by specific cytosine methylation in both strands. **b** Effect of strand-specific methylation of the 35S promoter on the *Fluc* expression. **c** Effect of the methylation of the cytosine residues (positions − 84, − 56 and − 33) at CHH context on the Fluc expression. Fluc activity was calibrated by Rluc activity. Values in the graph are shown as a fold change of the 35S-*Fluc* control (set to 1.0). Values are means with standard deviations obtained from three biological replicates. Data were analyzed using Tukey-Kramer test. Different lowercase letters at error bars indicate a significant difference between the means at the 5% level

To verify that the specific CG methylation could indeed compromise the gene expression under the 35S promoter, we developed a new method, in which we used the 105-bp 35S promoter sequence made by hybridizing two chemically synthesized oligonucleotides with methylated cytosine residues in a Golden gate cloning strategy (Fig. 7a). To evaluate the ability of the synthetic promoter to induce the downstream gene expression, we ligated it to the reporter gene firefly luciferase (Fluc), and Fluc activity was measured in protoplasts after transfection of the ligation products (Fig. 7b). As shown in Fig. 8a, Fluc activity was greatly reduced when both plus- and minus-strands were methylated at the cytosine positions − 45, − 46, − 65, − 66, − 77 and − 78. However, when either plus- or minus-strand was methylated, we found that methylation on the plus-strand barely suppressed Fluc activity and sometimes even increased it. In contrast, cytosine methylations at the minus-strand greatly reduced Fluc activity to 1/3 of the control, 35S-*Fluc* (Fig. 8b). Intriguingly, when we introduced both strands-methylated DNA, Fluc was suppressed at least to a level similar to that of the minus-strand or seemed to be more effective in the reduction of promoter activity, although the difference was not statistically significant (Fig. 8b).

### CHH methylation at a few cytosine residues in the subdomain A1 also contributed to reducing promoter activity

Because only three cytosine residues at CHH context (− 33, − 56, and − 84 in plus-sense) were found to be highly methylated within the domain A (− 90 ~ + 1) of the 35S promoter (Fig. 5a, Additional file 2: Figure S2a), we integrated methylated cytosine residues at those three CHH positions; two residues are located in the subdomain A1 and one residue is located in subdomain mp (region from − 45 to + 1). As shown in Fig. 8c, the introduced CHH methylation reduced the luciferase activity down to 1/3 of the control although the specific CG methylation tested was twice more effective (1/6 of the control).

## Discussion

### Unstable TGS phenotype in the progeny from VITGS-16c plants

In our analyses of RdDM on the 35S promoter induced by VITGS, we found discrete phenotypes of the TGS patterns against the *GFP* gene downstream of the 35S promoter. The TGS seemed to depend on the sizes of the 35S promoter sequence inserted in the viral vector. The TGS in the 16c plants inoculated with A1–345 was maintained in the S_1_ progeny, whereas we observed two phenotypes (RED and ORN) in the S_1_ progeny plants after inoculation with A1–208. The RED, from chlorophyll autofluorescence, represents a strong *GFP* TGS, and the ORN seems to be an intermediate TGS between those of 16c and the RED plants. In the S_1_ progeny from the plants inoculated with A1–116, most of the S_1_ plants lost the TGS of the *GFP* gene that had been observed in the virus-inoculated plants. Based on these observations, we considered that by analyzing the 35S promoters in these phenotypes, we might be able to reveal some important factor(s), which can control the robustness of the RdDM-mediated TGS.

We then analyzed the S2:208 plants in detail to obtain a clue about fixing a stable TGS for next generation. As shown in Fig. 4, the S2:208 ORN plants clearly had stronger GFP fluorescence in the stems and leaf veins (Vein) than in the leaf areas without veins (LwV). The *GFP* mRNA levels in the S2:208 ORN plants were significantly higher than in the S2:208 RED plants. Although we initially expected that the *GFP* levels in Vein would be higher than those in LwV, *GFP* expression differed little between the two tissues, perhaps because chlorophyll content in Vein tissues is lower than in LwV and the chlorophyll autofluorescence interfered less with the GFP fluorescence.

### Effect of cytosine methylation in the 35S promoter in a TSM assay on downstream gene expression

Here, we successfully developed an assay by which we can analyze the effect of specific cytosine methylation in a promoter sequence on the downstream gene expression. In addition, we can even discriminate DNA strand-sense when specific methylation is introduced into a target promoter. We here call this method strand-sense-specific methylation (triple S methylation, TSM) assay (Fig. 7). Previously, the promoter sequences that are important for TGS had been identified mainly using point-mutation and subdomain-deletion strategies [40–46]. However, without introducing actual methylation to specific cytosine residues, we can never conclude that methylation of the predicted sites is really responsible for the influence on the expression of downstream gene. Because we can directly manipulate cytosine methylation in a target promoter with a TSM assay, various promoter sequences can be analyzed to elucidate a link between specific cytosine methylation and promoter activities. The results of our TSM assays demonstrated that TGS levels of the 35S promoter might be differentially regulated depending on the heterogeneity of cytosine methylation in and between the plus- and minus-strands corresponding to the target domain sequences. Especially cytosine methylation at positions close to the *as-1* element (− 83 to − 63) is very important for stable, inherited TGS, which agrees well with the previously predicted cytosine residues for the promoter activity [40, 43, 45]. To be more specific, we found that TGS of the 35S promoter occurred efficiently when the cytosine residues (positions − 78, − 66 and − 46) on the minus-strand were methylated. On the other hand, surprisingly, the methylation of the corresponding sites on the plus-strand had little effect on the TGS, but the maximum TGS was observed when both strands were methylated at the specific cytosine residues. Our TSM assay also indicated that CHH methylation in the subdomain A1 significantly reduced promoter activity. However, considering that CHH methylation is not maintained through the next generation, the stable inherited TGS in the RED phenotype will be driven mainly by some specific symmetric methylations. No report has previously discussed the effect of heterogeneity of DNA methylation in and between the plus- and minus-DNA strands in relation to TGS. Although we have always assumed that both strands would be simultaneously and uniformly methylated in a model for RdDM [12], we should rather consider that the two strands may not be necessarily uniformly methylated in nature. Whether our observation for the 35S promoter can be generally applied to other promoters or not should be independently tested using a similar assay. Although in this study, we focused only on methylation of the subdomain A1, subdomains B2 and B4 in the 35S promoter have also been proved to be essential for promoter activity [46]. It would be worth evaluating the cytosine methylation on the other elements in the 35S promoter using the TSM assay.

### Link between symmetric and asymmetric methylation in inherited DNA methylation

In our bisulfite sequencing analyses, we noticed an interesting phenomenon that was always associated with the stable, inherited TGS in many replicates. In the RED plants in S2:208, we found that the overall levels of asymmetric (CHH) methylation were significantly reduced in both plus- and minus-strands, while the levels of symmetric methylation (CG and CHG) were conversely increased (Fig. 5 and Additional file 2: Figure S2). Most of the S_1_ plants generated from A1–116-inoculated plants lost TGS of the *GFP* gene. This unstable TGS in next generation may be associated with a decrease in symmetric methylation and an increase of asymmetric methylation in the target sequence. For cytosine methylation of the 35S promoter in the TGS-induced progeny plants (S_1_ to S_4_ generations), similar observations have also been shown in the study of the ALSV vector [24]. To understand the involvement of methylation status in TGS induction, we can test how the level of asymmetric methylation in the 35S promoter can affect TGS by a method like our TSM assay. Although we do not know the exact reason for a decrease in asymmetric methylation at this moment, it is conceivable that some factor during reproduction may have effects in the association between RdDM and inherited methylation. Considering the results together, we infer that the generation of the RED and ORN plants observed among the VITGS progeny plants may depend on the accumulation levels of symmetric methylation at some specific cytosine sites and a decrease in asymmetric methylation in the progeny plants.

## Conclusions

We induced TGS against the *GFP* gene under the 35S promoter through RdDM by a viral vector containing various sizes of the promoter sequence. In the first self-fertilized generation, the stable TGS seemed to be correlated with high levels of methylation on the promoter. However, we observed an unstable TGS in the second generation; S_2_ progenies were segregated into RED and ORN. Our bisulfite sequencing analyses suggested that specific methylation at a few cytosine residues in the subdomain A1 and a decrease in frequency of asymmetric methylation might be important for efficient induction of TGS through RdDM. To probe the importance of the methylation at specific cytosine residues, we developed a method by which we can analyze a direct effect of methylated cytosine residues on TGS in a strand-specific manner. In this assay, we found that cytosine methylation on either or both of the plus- and minus-sense promoter sequences could differentially drive gene expression downstream of the promoter. We therefore infer that robust TGS may be determined by the heterogeneity of DNA methylation at specific sites in and between the plus- and minus-strands.

## Methods

### Plant materials

*Nicotiana benthamiana* line 16c was obtained from Dr. David Baulcombe. The plants were grown with 16-h light (8-h dark) at 24 °C.

### Inoculation with virus and GFP observation

Fragments from the 35S promoter were amplified by PCR and inserted into the CMV-A1 vector. *N. benthamiana* line 16c plants were inoculated with in vitro transcripts of CMV as previously described by Otagaki et al. [36]. Total RNA was isolated 15 days postinoculation from the infected tissues. Viral cDNAs were PCR-amplified using a primer pair of 2b-5up and R2–2814-R2 (Additional file 5: Table S1) and the TaKaRa One Step RNA PCR Kit AMV (TaKaRa) kit according to the supplier’s instruction. To confirm that the viruses did not have serious mutations during their replication, the amplified viral cDNAs were directly sequenced: the sequencing chromatograms were shown in Additional file 6: Figure S5. GFP fluorescence was examined with a Keyence Microscope VB-7000 with the GFP filter (OP-42313).

### DNA methylation assay

DNA was extracted using the Illusta DNA extraction Kit PhytoPure (GE Healthcare) according to the supplier’s instructions. Bisulfite treatment of DNA was performed by using EZ DNA Methylation-Lightning Kit (Zymo Research) according to the supplier’s protocol. To amplify the target sequences, primer pair 35S-346F-bisuT/35S + 1A-bisuA were used for the plus-strand, and primer pair 35S (−)-5-BS/35S (−)-3-BS were used for the minus-strand (Additional file 5: Table S1). The primers were initially designed by the program, MethPrimer (http://www.urogene.org/cgi-bin/methprimer/methprimer.cgi). To amplify the entire (~ 345 bp) core sequence of the 35S promoter, we modified the designed primer so that they contained cytosine residues as less as possible, and synthesized degenerated primers. The PCR was conducted by Takara Epi-taq (TaKaRa) and the PCR conditions were as follows, 40 cycles of 94 °C for 30 s, 55 °C for 30 s and 72 °C for 30 s. The PCR products were then cloned into the pTAC1 vector using the Dyna Express TA PCR Cloning Kit (Bio Dynamics Laboratory). For clone-based bisulfite sequencing, we used 22–24 clones and the sequencing data were aligned using MEGA version 6 [47]. The graphs were created in Microsoft Excel (Additional file 7: Table S2).

### Quantitative RT-PCR analysis

Total RNA was isolated using TRIzol reagent (Invitrogen) according to the supplier’s instructions. For the DNA and RNA extraction in the S2 progenies, we separated class I and class II veins (Vein) [48] and leaf tissues without those veins (LwV). Extracted RNA was treated by DNase I recombinant RNase-free (Roche Applied Science), and then cDNA was synthesized from 0.2 μg of RNA using the PrimeScript RT reagent Kit (TaKaRa) according to the supplier’s instruction. qRT-PCR analysis was carried out by the Applied Biosystems StepOnePlus Real-time PCR system using the PowerUp SYBR Green Master Mix (Applied Biosystems). The PCR cycling conditions were 40 cycles of 95 °C for 15 s, 55 °C for 30 s and 72 °C for 30 s. The *GFP* expression levels were calculated using the comparative Ct method and normalized by the expression of the L23 gene. Target sequences were amplified using primer pair mGFP-5-160/mGFP3–160 for the *GFP* gene and primer pair Nb-L23–5-110/Nb-L23–3-110 for the internal control.

### Golden gate cloning and TSM assay

All oligonucleotides used in this study are listed in Additional file 5: Table S1. The unmethylated 35S promoter fragment was amplified from pBI221 (Clontech) by PCR. The target sequences were amplified using primer pair 35S-GGC-5/35S-GGC-3. The Fluc PCR product was also amplified by PCR using primer pair LUC-GGC-5/LUC-GGC-3. The custom-synthesized oligonucleotides (oligo DNAs) with specific methylation at three CG sites (positions − 78, − 66 and − 46) and at three CHH sites (positions − 33, − 56 and − 84) were prepared by the Custom DNA Synthesis Service (Hokkaido System Science). The oligo DNAs (100 μM) were annealed in oligo annealing buffer (10 mM Tris-HCl, 1 mM EDTA, 100 mM NaCl) with gradually cooling after incubation at 95 °C for 4 min. To make a DNA fragment where either strand was methylated, 35S-CG-DNA (+), 35S-CG-DNA (−) and 35S-CHH-DNA (+) were annealed with either unmethylated oligonucleotide 35S-3-120 PM or 35S-5-120 PM and then amplified by PCR. The PCR cycling conditions were 40 cycles of 94 °C for 30 s, 55 °C for 30 s and 72 °C for 30 s. All four types of DNA constructs were first digested by BsaI and then ligated to the firefly luciferase reporter (*Fluc*) gene as essentially described as golden gate cloning by Engler et al. [49]. We then analyzed the promoter activity in protoplasts. We call this method strand-sense-specific methylation (triple S methylation, TSM) assay.

### Protoplast transfection and luciferase assay

Protoplasts were prepared from the leaves of *N. benthamiana* as described by Shimura et al. [50]. Prepared Fluc-fused 35S promoter fragments (0.5 μg or 1 μg) were directly transfected to protoplasts together with pE-Rluc (0.45 μg) as an internal control in the presence of polyethylene glycol. After 18 h incubation, luciferase activities (Fluc and Rluc) were assayed using the Dual-Luciferase Reporter Assay System (Promega) and a fluorometer (Wallac 1420 ARVO MX) as previously described by Shimura et al. [50].

## Additional files

Additional file 1: **Figure S1.** Methylation status in the plus-strand of the 35S promoter of the 16c plants. Methylation frequency in the plus-strand of the 35S promoter in the Vein and LwV tissues was analyzed by bisulfite sequencing. *N* is the number of the clones used for sequencing. The *x*-axis is the position relative to the nucleotide distance from the transcription start sites. (TIFF 2598 kb)

Additional file 2: **Figure S2.** Cytosine methylation frequency in the 35S promoter in the LwV tissues of S2:208 RED and S2:208 ORN. a Comparison of methylation status in the plus-sense of the 35S promoter between S2:208 RED and S2:208 ORN. b Summary of the results in a to show differences in CHH, CHG, CG and total methylation. c Comparison of methylation status in the minus-sense of the 35S promoter between S2:208 RED and ORN. Asterisk indicates cytosine residue that is significantly different in methylation frequency between RED and ORN as explained in Additional file 3: Figure S3b. d Summary of the results in c to show differences in CHH, CHG, CG and total methylation. *N* is the number of the clones used for the bisulfite sequencing. The *x*-axis shows the position relative to the transcription start site (+1). The asterisks in b and d indicate a statistical significance in methylation frequencies by two-tailed Fisher’s exact test (* *P* < 0.05, ** *P* < 0.01). (TIFF 6706 kb)

Additional file 3: **Figure S3.** Difference in specific cytosine methylation between S2:208 RED and S2:208 ORN LwV tissues. a Difference in methylation frequencies in the overall plus- and minus-strand of the 35S promoter in S2:208 RED and S2:208 ORN. To make it easier to find any differences in methylation frequencies in each strand, values of S2:208 RED LwV (Additional file 2: Figure S2a and c, upper graphs) were subtracted from those of S2:208 ORN LwV (Additional file 2: Figure S2a and c, lower graphs). b Close-up of the subdomain A1 in a. The *x*-axis is the nucleotide sequence of the subdomain A1. Nucleotide sequences of the plus- and minus-strand of the subdomain A1 are indicated below the graph. Methylated CG and CHG sites are underlined. The asterisk in b indicates a statistical significance between S2:208 RED and S2:208 ORN by two-tailed Fisher’s exact test (* *P* < 0.05). (TIFF 7061 kb)

Additional file 4: **Figure S4.** Methylation status in the plus-strand of the 35S promoter in transgenic plants. a, b Methylation frequencies of GFP-silenced lines. Lines 2–6–1-7-6 (a) and 2–6–1-7-2 (b) were the T_5_ progeny lines derived from the original line 2–6–1-7, which contained a direct repeat of the 35S promoter followed by the *GFP* gene sequence. *GFP* expression in 2–6–1-7 and 2–6–1-7-6 initially decreased as a result of post-transcriptional gene silencing (PTGS) and later by TGS, while *GFP* expression in 2–6–1-7-2 was stably suppressed by TGS. Twelve to fifteen clones were used for the bisulfite sequencing. c Values for a were subtracted from those for b to show differences in methylation frequencies between 2 and 6–1-7-2 and 2–6–1-7-6. The positions − 82, − 78, − 66 and − 46 indicated specific cytosine residues located in the subdomain A1. The *x*-axis shows the position relative to the transcription start site (+ 1). Positions of the subdomains A1, B2 and B4 are also indicated. (TIFF 4447 kb)

Additional file 5: **Table S1.** List of primers used in this study. (XLSX 10 kb)

Additional file 6: **Figure S5.** 35S promoter sequences in the viral genomes of the vector isolated from infected tissues. a-c Sequencing chromatograms of the inserts containing each of 345-, 208- and 116-bp portion (a to c, respectively). Total RNA was isolated 15 days postinoculation, and RT-PCR-amplified fragment were directly sequenced. We confirmed that the original sequences integrated into the viral vector did not change in the infected tissues. (TIFF 7535 kb)

Additional file7: **Table S2.** Raw data of our clone-based bisulfite sequencing used for Figs. 3 and 5, Additional files 1, 2, 3 and 4: Figures S1–S4. All the graphs for methylation status were calculated in those Excel files. (XLSX 347 kb)

## Abbreviations

AGO: Argonaute
ALSV: Apple latent spherical virus
CMT2: CHROMOMETHYLASE2
CMT3: CHROMOMETHYLASE3
CMV: Cucumber mosaic virus
DCL3: DICER-LIKE 3
DRM2: DOMAINS REARRANGED METHYLTRANSFERASE2
dsRNA: double stranded RNA
GFP: Green fluorescent protein
MET1: METHYLTRANSFERASE1
NRPE1: NUCLEAR RNA POLYMERASE E1
PTGS: Post-transcriptional gene silencing
RdDM: RNA directed DNA methylation
RDR2: RNA dependent RNA polymerase 2
siRNA: short interfering RNA
TGS: Transcriptional gene silencing
VITGS: Virus-induced transcriptional gene silencing
