# Supplementary figures and images for: Transcriptional silencing of 35S driven-transgene is differentially determined depending on promoter methylation heterogeneity at specific cytosines in both plus- and minus-sense strands
Source: BMC Plant Biol. 2019 Jan 14;19:24. doi: 10.1186/s12870-019-1628-y (PMC6332629; doi:10.1186/s12870-019-1628-y)

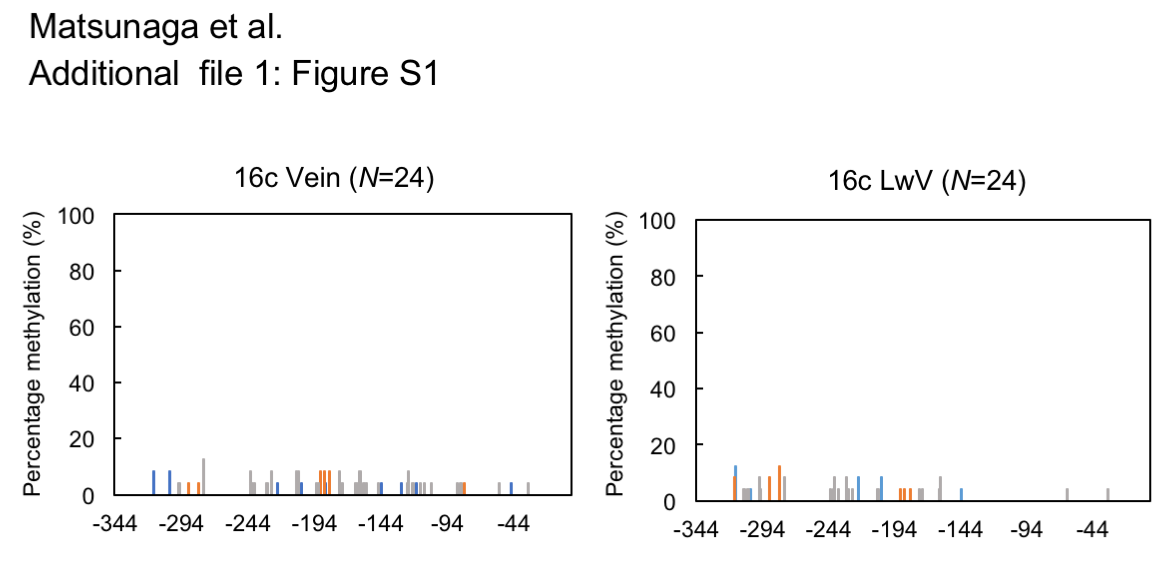

Supplement: Supplementary file 1 — Figure S1. Methylation status in the plus-strand of the 35S promoter of the 16c plants. Methylation frequency in the plus-strand of the 35S promoter in the Vein and LwV tissues was analyzed by bisulfite sequencing. N is the number of the clones used for sequencing. The x-axis is the position relative to the nucleotide distance from the transcription start sites. (TIFF 2598 kb) [file 12870_2019_1628_MOESM1_ESM.tiff]

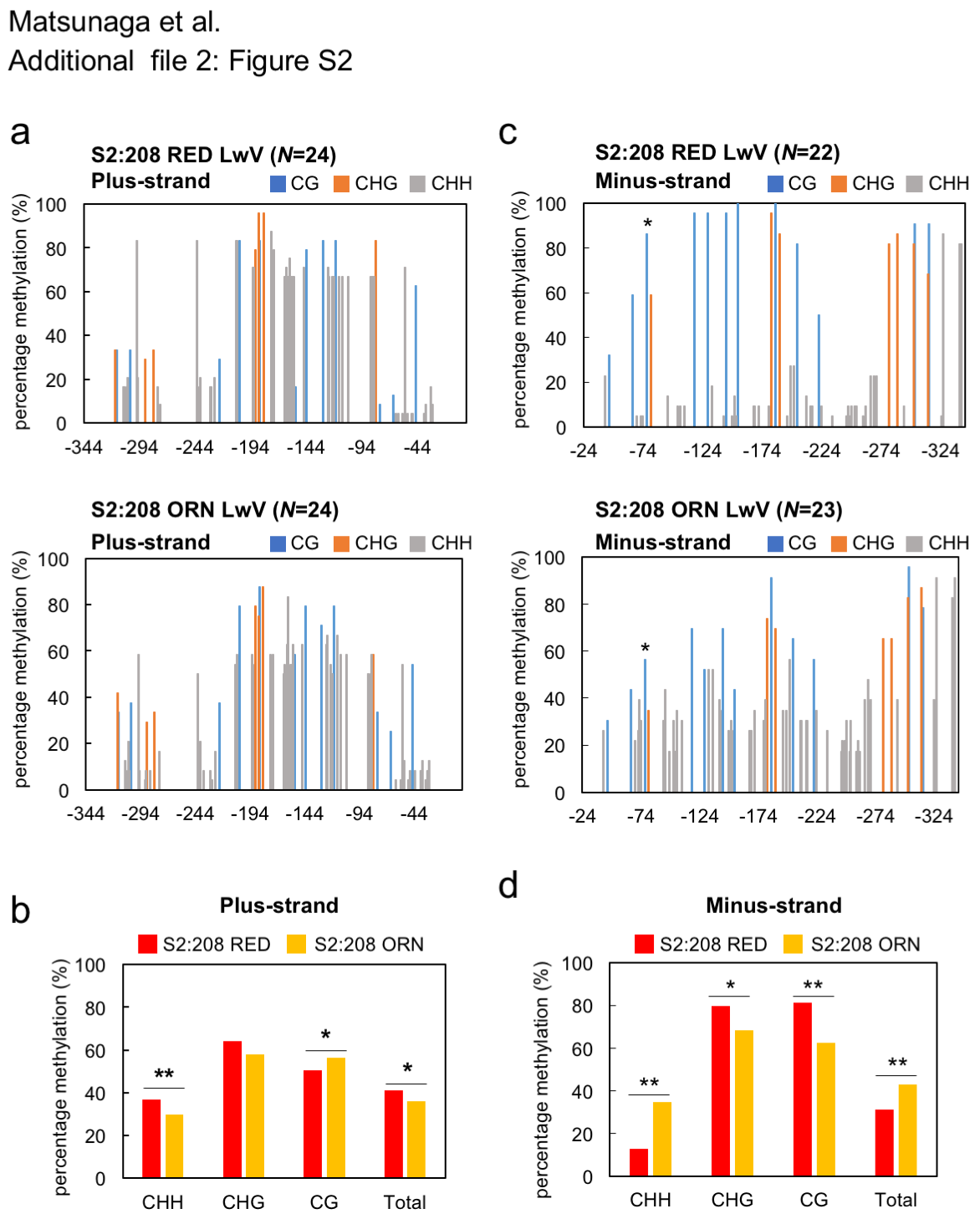

Supplement: Supplementary file 2 — Figure S2. Cytosine methylation frequency in the 35S promoter in the LwV tissues of S2:208 RED and S2:208 ORN. a Comparison of methylation status in the plus-sense of the 35S promoter between S2:208 RED and S2:208 ORN. b Summary of the results in a to show differences in CHH, CHG, CG and total methylation. c Comparison of methylation status in the minus-sense of the 35S promoter between S2:208 RED and ORN. Asterisk indicates cytosine residue that is significantly different in methylation frequency between RED and ORN as explained in Additional file 3: Figure S3b. d Summary of the results in c to show differences in CHH, CHG, CG and total methylation. N is the number of the clones used for the bisulfite sequencing. The x-axis shows the position relative to the transcription start site (+1). The asterisks in b and d indicate a statistical significance in methylation frequencies by two-tailed Fisher’s exact test (* P < 0.05, ** P < 0.01). (TIFF 6706 kb) [file 12870_2019_1628_MOESM2_ESM.tiff]

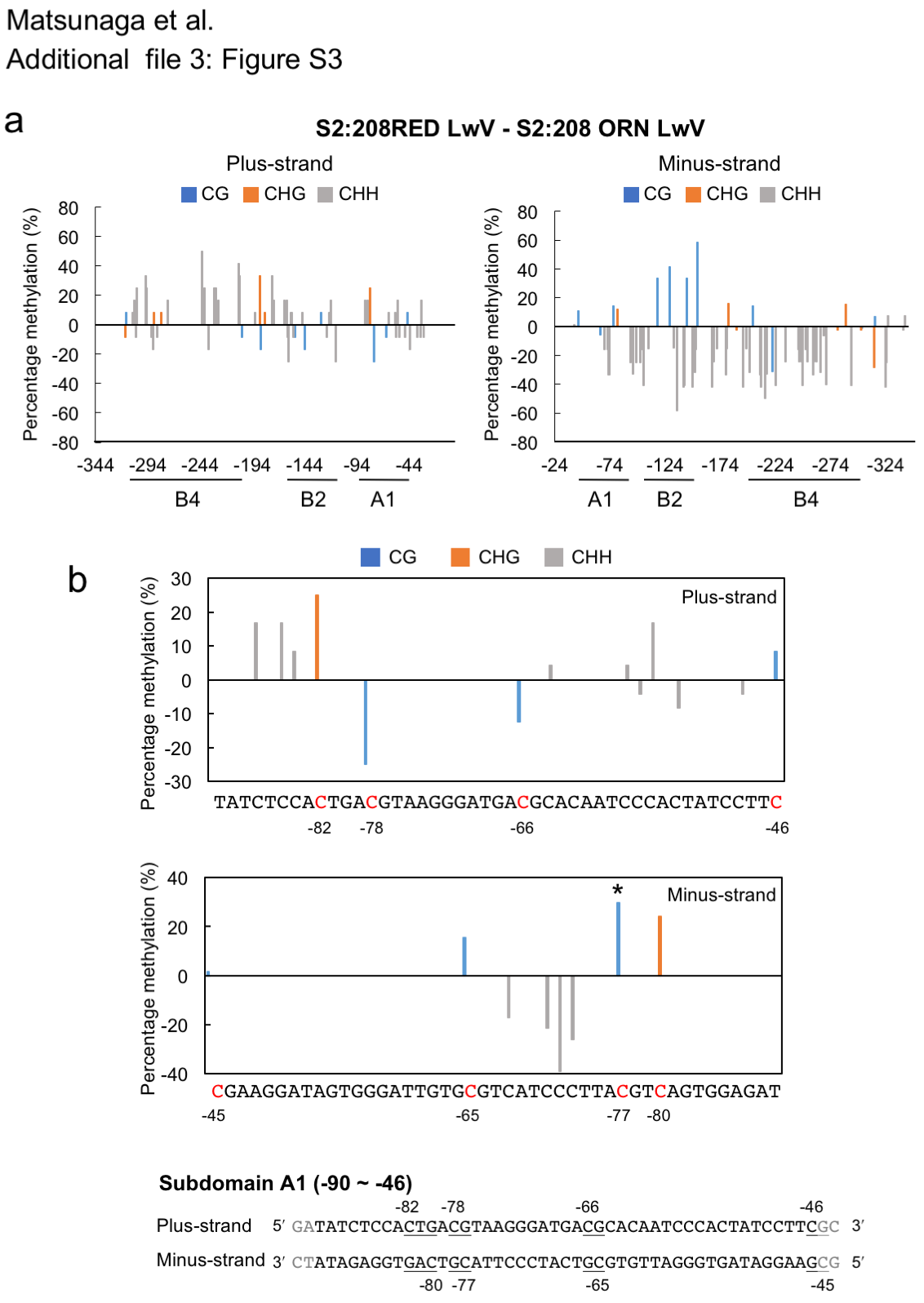

Supplement: Supplementary file 3 — Figure S3. Difference in specific cytosine methylation between S2:208 RED and S2:208 ORN LwV tissues. a Difference in methylation frequencies in the overall plus- and minus-strand of the 35S promoter in S2:208 RED and S2:208 ORN. To make it easier to find any differences in methylation frequencies in each strand, values of S2:208 RED LwV (Additional file 2: Figure S2a and c, upper graphs) were subtracted from those of S2:208 ORN LwV (Additional file 2: Figure S2a and c, lower graphs). b Close-up of the subdomain A1 in a. The x-axis is the nucleotide sequence of the subdomain A1. Nucleotide sequences of the plus- and minus-strand of the subdomain A1 are indicated below the graph. Methylated CG and CHG sites are underlined. The asterisk in b indicates a statistical significance between S2:208 RED and S2:208 ORN by two-tailed Fisher’s exact test (* P < 0.05). (TIFF 7061 kb) [file 12870_2019_1628_MOESM3_ESM.tiff]

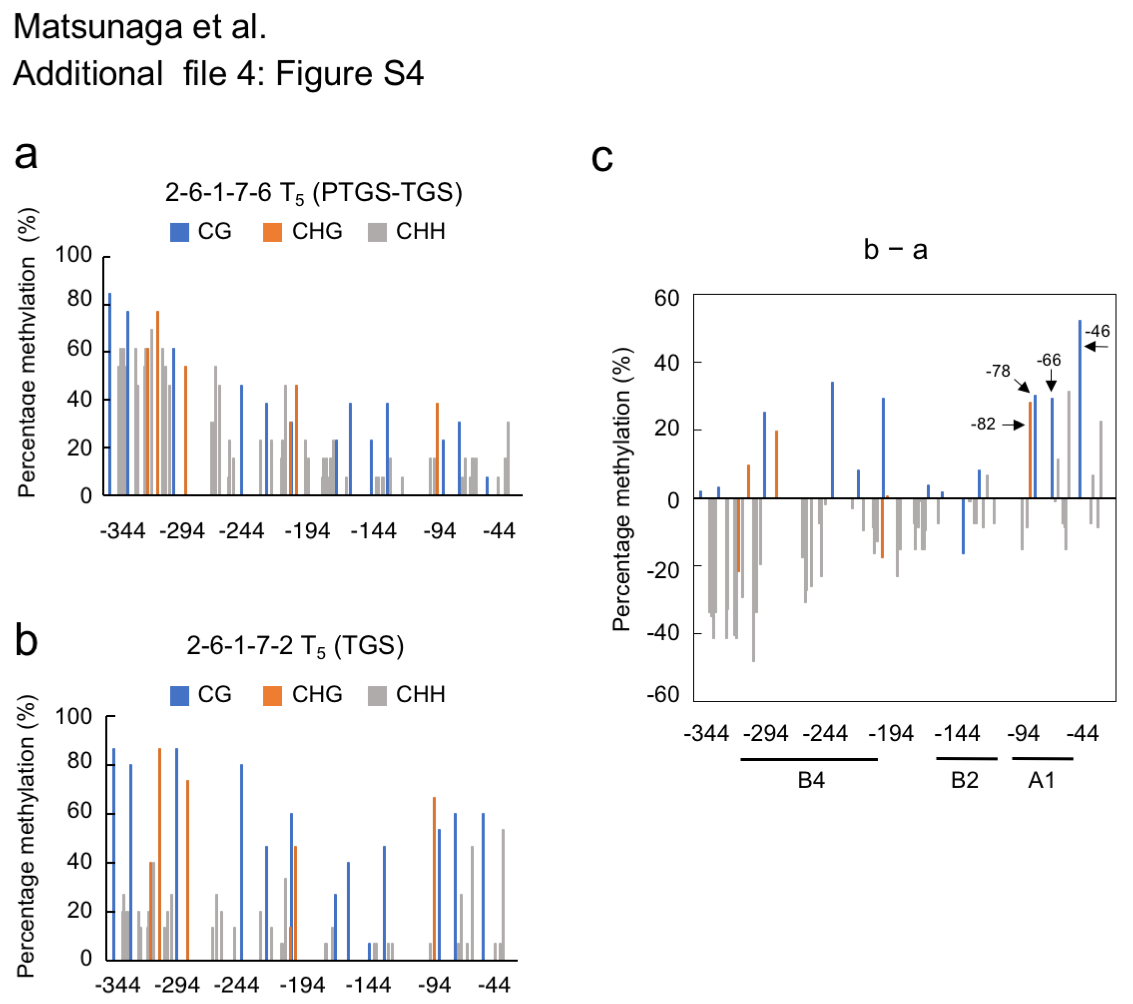

Supplement: Supplementary file 4 — Figure S4. Methylation status in the plus-strand of the 35S promoter in transgenic plants. a, b Methylation frequencies of GFP-silenced lines. Lines 2–6–1-7-6 (a) and 2–6–1-7-2 (b) were the T5 progeny lines derived from the original line 2–6–1-7, which contained a direct repeat of the 35S promoter followed by the GFP gene sequence. GFP expression in 2–6–1-7 and 2–6–1-7-6 initially decreased as a result of post-transcriptional gene silencing (PTGS) and later by TGS, while GFP expression in 2–6–1-7-2 was stably suppressed by TGS. Twelve to fifteen clones were used for the bisulfite sequencing. c Values for a were subtracted from those for b to show differences in methylation frequencies between 2 and 6–1-7-2 and 2–6–1-7-6. The positions − 82, − 78, − 66 and − 46 indicated specific cytosine residues located in the subdomain A1. The x-axis shows the position relative to the transcription start site (+ 1). Positions of the subdomains A1, B2 and B4 are also indicated. (TIFF 4447 kb) [file 12870_2019_1628_MOESM4_ESM.tiff]

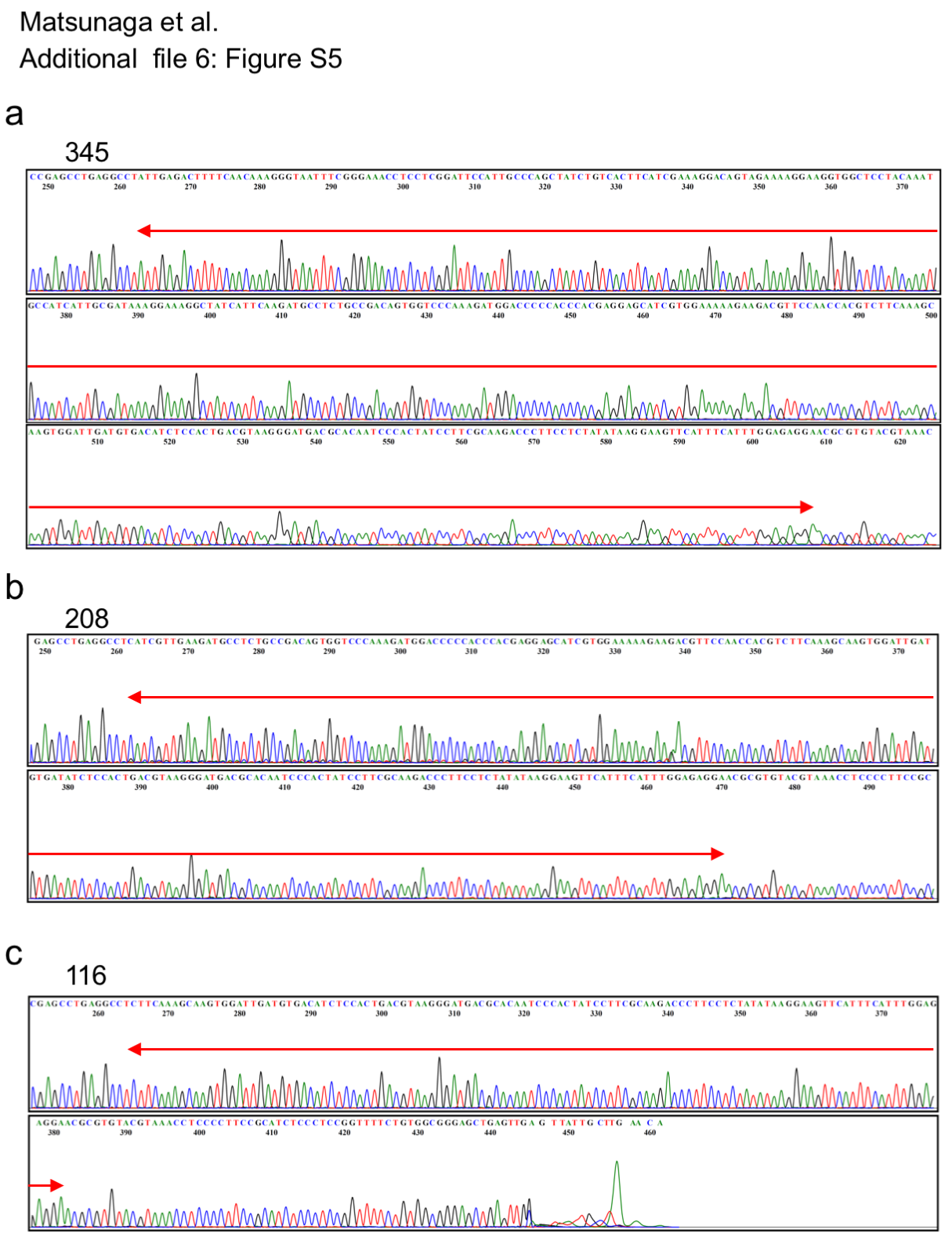

Supplement: Supplementary file 6 — Figure S5. 35S promoter sequences in the viral genomes of the vector isolated from infected tissues. a-c Sequencing chromatograms of the inserts containing each of 345-, 208- and 116-bp portion (a to c, respectively). Total RNA was isolated 15 days postinoculation, and RT-PCR-amplified fragment were directly sequenced. We confirmed that the original sequences integrated into the viral vector did not change in the infected tissues. (TIFF 7535 kb) [file 12870_2019_1628_MOESM6_ESM.tiff]
